# Catalytic Activity of cGMP-Dependent Protein Kinase Type I in Intact Cells Is Independent of N-Terminal Autophosphorylation

**DOI:** 10.1371/journal.pone.0098946

**Published:** 2014-06-04

**Authors:** Raghavan Vallur, Hubert Kalbacher, Robert Feil

**Affiliations:** 1 Interfakultäres Institut für Biochemie, University of Tübingen, Tübingen, Germany; 2 German Center for Neurodegenerative Diseases (DZNE), Tübingen, Germany; 3 Graduate School of Cellular & Molecular Neuroscience, University of Tübingen, Tübingen, Germany; University of Oldenburg, Germany

## Abstract

Although cGMP-dependent protein kinase type I (cGKI) is an important mediator of cGMP signaling and upcoming drug target, its *in vivo*-biochemistry is not well understood. Many studies showed that purified cGKI autophosphorylates multiple sites at its N-terminus. Autophosphorylation might be involved in kinase activation, but it is unclear whether this happens also in intact cells. To study cGKI autophosphorylation *in vitro* and *in vivo*, we have generated phospho-specific antisera against major *in vitro*-autophosphorylation sites of the cGKI isoforms, cGKIα and cGKIβ. These antisera detected specifically and with high sensitivity phospho-cGKIα (Thr58), phospho-cGKIα (Thr84), or phospho-cGKIβ (Thr56/Ser63/Ser79). Using these antisera, we show that ATP-induced autophosphorylation of cGKI in purified preparations and cell extracts did neither require nor induce an enzyme conformation capable of substrate heterophosphorylation; it was even inhibited by pre-incubation with cGMP. Interestingly, phospho-cGKI species were not detectable in intact murine cells and tissues, both under basal conditions and after induction of cGKI catalytic activity. We conclude that N-terminal phosphorylation, although readily induced *in vitro*, is not required for the catalytic activity of cGKIα and cGKIβ *in vivo*. These results will also inform screening strategies to identify novel cGKI modulators.

## Introduction

Cyclic guanosine monophosphate (cGMP) acts as a second messenger in various cell types and tissues of eukaryotes [1], [2]. The intracellular concentration of cGMP depends on the rate of its synthesis and degradation. cGMP is generated by cytosolic soluble guanylyl cyclases in response to NO or by membrane-bound particulate guanylyl cyclases that are activated by natriuretic peptides and some bacterial toxins. cGMP is hydrolyzed to GMP by phosphodiesterases, whose catalytic activity is often regulated by binding of cGMP or cAMP. At least three classes of cGMP effector proteins are known: cyclic nucleotide-gated cation channels, which transduce changes in cGMP concentrations into changes of membrane potential; cGMP-regulated cAMP-hydrolyzing phosphodiesterases, which mediate a cross-talk of cGMP and cAMP signaling; and cGMP-dependent protein kinases, which upon binding of cGMP phosphorylate a variety of target proteins at Ser/Thr residues.

The cGMP-dependent protein kinase type I (cGKI, also known as PKG-I or PRKG1) is considered a major mediator of cGMP signaling in mammals. Many studies suggest that pharmacologic regulation of cGKI might interfere with diverse patho-physiological processes [3], [4]. Thus, small-molecule modulators of cGKI for *in vivo*-use are of great interest to basic and clinical research. However, the development of such drugs has been hampered, in part, because the *in vivo*-biochemistry of cGKI is not well understood.

**Figure 1:**
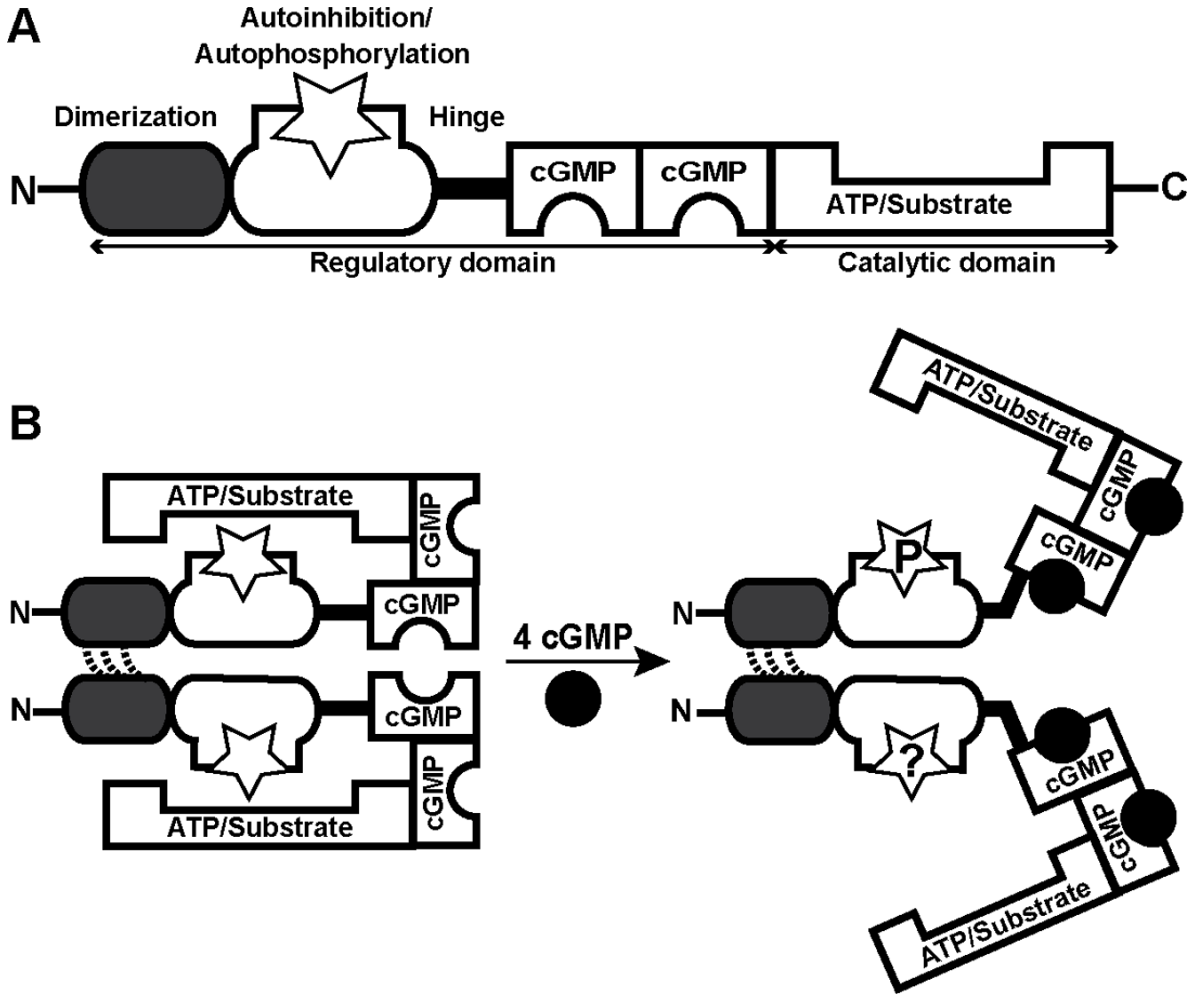
General structure and current working model of cGKI. (A) cGKI consists of a C-terminal catalytic domain and an N-terminal regulatory domain. The catalytic domain contains binding sites for ATP and protein substrates with Ser/Thr residues. The regulatory domain comprises two non-identical cGMP-binding pockets and additional regions with multiple functions: a leucine zipper for dimerization of two identical subunits, an overlapping autoinhibitory/autophosphorylation region (open star), and a flexible hinge region connecting the N-terminal region to the rest of the protein. (B) According to the current model, the homodimeric enzyme cannot heterophosphorylate substrates in the absence of cGMP (*left*). Binding of cGMP (black circles) results in a conformational change that allows heterophosphorylation of substrates (*right*). According to *in vitro* studies with purified cGKI, the N-terminal region of the inactive kinase is not phosphorylated (*left*, stars), and activation is associated with autophosphorylation of distinct sites in this region (*right*, star with a “P”). However, it is not clear whether or not N-terminal phosphorylation of cGKI does also occur in intact cells (*right*, star with a “?”).

cGKI is composed of an N-terminal regulatory domain that contains two non-identical cGMP-binding pockets with different affinities for cGMP and a C-terminal catalytic domain with binding sites for ATP and protein substrates [5]–[7] (Fig. 1A). The mammalian *prkg1* gene encodes two cGKI isoforms, cGKIα and cGKIβ. Each isozyme forms a homodimer of two ≈75 kDa subunits. cGKIα and cGKIβ have identical cGMP-binding and catalytic domains, but differ in their N-terminal regions (≈100 amino acids). This region mediates dimerization via a leucine zipper motif, regulates the affinity of the cGMP-binding pockets via allosteric mechanisms, and interacts, presumably in an isoform-specific manner, with anchoring and substrate proteins. It also contains an autoinhibitory/autophosphorylation region that might be involved in enzyme activation (Fig. 1).

Experiments conducted about 35 years ago revealed that purified cGKI undergoes autophosphorylation of its N-terminal region in the presence of radioactively labeled Mg^2+^-ATP [8]–[10]. In cGKIα, major *in vitro*-autophosphorylation sites were identified as Ser50, Thr58, Ser72, and Thr84, and a lower extent of autophosphorylation was observed on Ser1, Ser26, Ser44, and Ser64 [11]–[13]. In cGKIβ, Ser63 and Ser79 were identified as *in vitro*-autophosphorylation sites [14], [15]. Note that, in these reports, the numbering system used to identify amino acid residues omitted the N-terminal Met. This nomenclature was also adopted in the present study.

According to the current working model of cGKI (Fig. 1B), binding of cGMP induces a conformational change that releases the inhibition of the catalytic domain by the autoinhibitory region, perhaps via autophosphorylation of the autoinhibitory region [5], [6]. Autophosphorylation of certain sites increases basal phosphotransferase activity and the affinity for cGMP, but it can also promote the degradation of the enzyme [16]. Moreover, the introduction of phosphates in the N-terminal region could influence the interactions of cGKI isoforms with other proteins. Taken together, the previous *in vitro* studies indicated that N-terminal autophosphorylation regulates various biochemical properties of cGKI, with both positive and negative effects on cGMP signaling. Pharmacologic modulation of cGKI autophosphorylation, possibly in an isoform-specific manner, might therefore be a promising strategy to influence cGMP signaling *in vivo*. However, very little is known about the relevance of cGKI autophosphorylation *in vivo* in intact cells and tissues [5].

In the present study, phospho-specific antibodies were generated that detect autophosphorylated cGKIα and cGKIβ with high sensitivity. Our results indicate that N-terminal autophosphorylation of cGKI does readily occur in purified protein preparations or cell extracts but not in intact cells.

## Materials and Methods

### Ethics statement

All animal procedures were in compliance with the European Community guidelines for the use of experimental animals and had been approved by the committee on animal care and welfare of the Regierungspräsidium Tübingen, Baden-Württemberg, Germany.

### Materials

cGMP, 8-Br-cGMP, 8-Br-PET-cGMP, and 8-Br-cAMP were purchased from Biolog Life Sciences Institute. C-type natriuretic peptide, ATP, calyculin A, isoprenaline hydrochloride, and DEA-NONOate were from Abbiotec, AppliChem, Cell Signaling, Sigma-Aldrich, and ENZO Life Sciences, respectively. PhosSTOP phosphatase inhibitor cocktail tablets were from Roche.

### Peptide synthesis and antibody generation

Peptides containing known N-terminal cGKI autophosphorylation sites were selected based on the murine proteins (NP_001013855.1 for cGKIα and NP_035290.1 for cGKIβ) and synthesized with and without the corresponding phospho-amino acids. The peptides were synthesized as single peptides and as multiple antigen peptides, (peptide)_8_-(Lys)_4_-(Lys)_2_-Lys-β-Ala-OH, using standard Fmoc/tBu chemistry [17] on a multiple peptide synthesizer, Syro II (MultiSynTech, Witten, Germany). The single peptides were purified using reversed phase-HPLC and their identity was confirmed using ESI-MS and MALDI-TOF-MS. Peptide purities were >95% as determined by analytical reversed phase-HPLC. The single peptides were coupled to keyhole limpet hemocycanin using the glutardialdehyde method. The antisera were obtained after repeated immunization of rabbits with a 1: 1-mixture of the peptide–keyhole limpet hemocycanin conjugate and the multiple antigen peptide. A total of seven antigenic phospho-peptides were injected (Pineda Antibody-Service GmbH). As depicted in Table 1, 3 peptide pools (I, II, III; each containing 2–3 peptides) were injected into 9 rabbits (3 rabbits per pool) to obtain 9 polyclonal antisera (PS 1–9).

**Table 1:** Phospho-peptides used to generate antibodies against phospho-cGKI species.

cGKI	Peptide	Peptide	Peptide	Rabbit
isozyme	pool	ID	sequence	antiserum
Iα	I	Iα S50	SVLPVPpSTHIG	1–3
		Iα T58	GPRTpTRAQGISA	
Iα	II	Iα S72	AEPQTYRpSFHDLRQA	4–6
		Iα T84	RQAFRKFpTKSERSK	
Iβ	III	Iβ T56	IRPApTQQAQK	7–9
		Iβ S63	QAQKQpSASTL	
		Iβ S79	RTKRQAIpSAEPTA	

Fragments of the N-terminal regions containing *in vitro*-autophosphorylation sites of cGKIα and cGKIβ were synthesized with phosphorylated residues at the indicated positions (pS, phospho-Ser, pT, phospho-Thr). Three peptide pools (I–III), each containing 2 or 3 phospho-peptides, were used to immunize rabbits (3 rabbits per peptide pool). Peptide sequences were derived from murine cGKIα and cGKIβ, and the amino acids were numbered without counting the N-terminal Met.

Affinity purification of antiserum PS3 was performed as described previously [18]. 10 mg of phospho- or nonphospho-peptide (GPRTpTRAQGISA or GPRTTRAQGISA, respectively, where pT indicates phospho-Thr) were coupled separately to 1 g of CH-activated Sepharose (GE Healthcare) according to the manufacturer's instructions. First, 5 ml of the antiserum was diluted with 5 ml phosphate-buffered saline (PBS) and applied to the phospho-peptide Sepharose column (10×1 cm). The column was rotated overnight at 4°C, washed thoroughly with PBS, and then the bound antibodies were eluted with 0.1 M citrate buffer (pH 3.0) and immediately neutralized with 0.5 M phosphate buffer (pH 8.0). Then, the eluate was applied to the nonphospho-peptide Sepharose column (10×1 cm). After overnight rotation at 4°C, the flow-through was collected and concentrated to 0.4 mg protein/ml using a 20 kD ultrafiltration membrane (Amicon). The affinity purified PS3 serum was abbreviated as AffPS3.

### Enzyme-linked immunosorbent assay (ELISA)

ELISAs were performed as described [17]–[19]. Wells of 96-well plates (MaxiSorb surface, Nunc Brand products, Wiesbaden, Germany) were coated with phospho- or nonphospho-peptides (10 µg in 100 µl PBS/well) (Table 1) overnight at 4°C in an orbital shaker. Wells were washed three times with wash buffer (0.05% Tween 20 in PBS, pH 7.0) and then incubated with 2% bovine serum albumin in wash buffer for 2 h at 37°C. After three washes, wells were incubated for 1.5 h at 37°C with non-purified or purified antisera (diluted 1∶20000 or 1∶100, respectively, in wash buffer containing 0.5% bovine serum albumin). After five washes, wells were incubated with horseradish peroxidase-conjugated goat anti-rabbit IgG (Dianova) for 1 h at 37°C (1∶2000 diluted in wash buffer containing 0.5% bovine serum albumin). After five washes with wash buffer, 100 µl of 1 mg/ml azino-diethylbenzthiazoline sulfonate/H_2_O_2_ in 0.1 M citrate buffer (pH 4.5) were added to each well. After 20 min at 37°C, light absorbance was measured at 405 nm.

### Autophosphorylation of purified cGKI isozymes

Recombinant bovine cGKIα and cGKIβ were expressed in Sf9 insect cells and purified by affinity chromatography as described [20], [21]. Purified proteins were incubated at 30°C for 15 min in a total volume of 80 µL. The reaction mix contained 50 mM Mes, 0.4 mM EGTA, 1 mM magnesium acetate, 10 mM NaCl, 10 mM dithiothreitol, and 8 µg cGKIα or cGKIβ. Autophosphorylation was initiated by adding 0.1 mM ATP or 0.1 mM ATP combined with 0.1 mM cGMP. In some experiments, aliquots of the reaction mixtures were pre-incubated with cGMP or subsequently treated with lambda protein phosphatase (200 units/µg protein; NEB) at 30°C for 90 min. The reactions were stopped by adding 1x SDS-PAGE loading buffer and heating for 5 min at 95°C. Samples were stored at −20°C.

### Cell culture

Wild-type and cGKI-deficient (genotype: cGKI^L-/L-^) [22] mouse embryonic fibroblasts (MEFs) and primary vascular smooth muscle cells (VSMCs) were obtained as described [23], [24]. All cells were from mice on a 129/Sv genetic background. They were cultured in Dulbecco's modified Eagle medium (DMEM) supplemented with 10% fetal calf serum, 100 U/mL penicillin, and 100 mg/mL streptomycin at 37°C and 6% CO_2_. MEFs were used for experiments between passage 20 and 39. VSMCs were isolated from aortae of 5- to 6-week-old mice and analyzed in primary culture without passaging.

### Phosphorylation of cGKI in intact cells and tissues

Cultured cells were serum-starved (MEFs for 3 h and VSMCs for 48 h) in DMEM containing 100 U/mL penicillin and 100 mg/mL streptomycin at 37°C and 6% CO_2_. Then, test compounds were added in PBS in the absence or presence of 100 nM calyculin A for various times at 37°C and 6% CO_2_ as specified in the respective figure legends. At the end of the treatment, cells were washed twice with ice-cold PBS, lysed in lysis buffer A (21 mM Tris-Cl, pH 8.3, 0.7% SDS, 0.2 mM phenylmethylsulfonyl fluoride, and one PhosSTOP tablet per 10 mL) and heated for 5 min at 95°C. Samples were stored at −20°C.

Native tissues (aorta, lung, bladder, cerebellum) were isolated from 4- to 12-week-old wild-type and cGKI-deficient mice (genotype:cGKI^L-/L-^) [22] on a 129/Sv or C57BL/6 genetic background. Mice were sacrificed by cervical dislocation. For the analysis of basal cGKI phosphorylation, tissues were rapidly dissected in ice-cold PBS, snap frozen in liquid N_2_ and stored at −70°C. To evaluate the effects of various agents on cGKI phosphorylation, tissues were rapidly dissected and then incubated with the test compounds in Tyrode buffer (5 mM HEPES, pH 7.4, 140 mM NaCl, 5 mM KCl, 1.2 mM MgSO_4_, 2.5 mM CaCl_2_, 5 mM Glucose) in the absence or presence of 100 nM calyculin A as specified in the respective figure legends. For Western blot analysis, tissue homogenates were prepared in lysis buffer B (50 mM Tris-Cl, pH 8.3, 100 mM NaCl, 2% SDS, 5 mM EDTA, 2.5 mM phenylmethylsulfonyl fluoride, and one PhosSTOP tablet per 10 mL) using a FastPrep homogenizer (lysing matrix A; MP Biomedicals). Samples were heated for 5 min at 95°C and stored at −20°C.

Platelets were isolated from 8- to 12-week-old wild-type mice on a C57BL/6 genetic background as described [25]. The platelets were incubated for 60 min at room temperature, then for 10 min at 37°C, and then they were treated with drugs for 15 min at 37°C. Subsequently, they were lysed by adding 1x SDS-PAGE loading buffer. Samples were heated for 5 min at 95°C and stored at −20°C.

### Phosphorylation of cGKI in cell extracts

MEFs were serum-starved for 3 h in DMEM containing 100 U/mL penicillin and 100 mg/mL streptomycin at 37°C and 6% CO_2_. Using a cell scraper, cells were harvested in ice-cold buffer C (20 mM Tris, pH 8.3, 100 mM NaCl, 0.2 mM phenylmethylsulfonyl fluoride, and one PhosSTOP tablet per 10 mL). The cell suspension was subjected to sonication (Bandelin SONOPLUS; 4×30 s on ice, with a power of 55% and 30 s rest in between the cycles) followed by centrifugation at 18,000 g for 10 min at 4°C. The supernatant was collected and the protein concentration was measured with the Bradford assay.

To induce phosphorylation of cGKI, MEF extracts were incubated at 30°C for 15 min in a total volume of 500 µL. The reaction mix contained 50 mM Mes, 0.4 mM EGTA, 1 mM magnesium acetate, 10 mM NaCl, 10 mM dithiothreitol, and 200 µg cell extract. Phosphorylation was initiated by adding 0.1 mM ATP or 0.1 mM ATP combined with 0.1 mM cGMP. In some experiments, the reaction mixtures were pre-incubated with cGMP before ATP was added. The reactions were stopped by adding 1x SDS-PAGE loading buffer and heating for 5 min at 95°C. Samples were stored at −20°C.

### Western blot analysis

Proteins were separated by SDS-PAGE and blotted on polyvinylidene difluoride membranes (Millipore). Antibodies used were rabbit anti-cGKI common (DH) (1∶5000), a pan-specific (nonphospho-specific) antiserum detecting both cGKIα and cGKIβ [26], rabbit anti-VASP (1∶1000, Cell Signaling, 9A2, 3132), rabbit anti-GAPDH (1∶5000, Cell Signaling, 14C10, 2118), and rabbit anti-Akt (1∶1000; Cell Signaling, 9272). The polyclonal rabbit antisera against phospho-cGKI species that were generated and characterized in this study were AffPS3 (1∶100), PS6 (1∶2000), and PS7 (1∶2000). According to the detected phospho-site(s), AffPS3, PS6, and PS7 are also designated as anti-cGKIα (phospho-Thr58), anti-cGKIα (phospho-Thr84), and anti-cGKIβ (phospho-Thr56, phospho-Ser63, phospho-Ser79), respectively. As secondary antibody, goat anti-rabbit horseradish peroxidase-conjugated IgG (1∶3000; Cell Signaling, 7074) was used.

### Statistics

Data are expressed as mean ± SEM. Significance was determined by using Student's *t* test.

## Results

### The phospho-specific antisera detect autophosphorylated cGKI isoforms with high specificity and sensitivity

To detect N-terminally phosphorylated cGKI species, we sought to generate phospho-specific rabbit polyclonal antisera against the major *in vitro*-autophosphorylation sites reported previously: phospho-Ser50, phospho-Thr58, phospho-Ser72, and phospho-Thr84 in cGKIα [11]–[13] and phospho-Ser63 and phospho-Ser79 in cGKIβ [14], [15]. The potential for phosphorylation of these sites was confirmed *in silico* by a group-based phosphorylation predicting and scoring method [27]. The *in silico* method did also identify Thr56 of cGKIβ as a potential autophosphorylation site and, therefore, this site was also selected for antibody generation.

**Figure 2:**
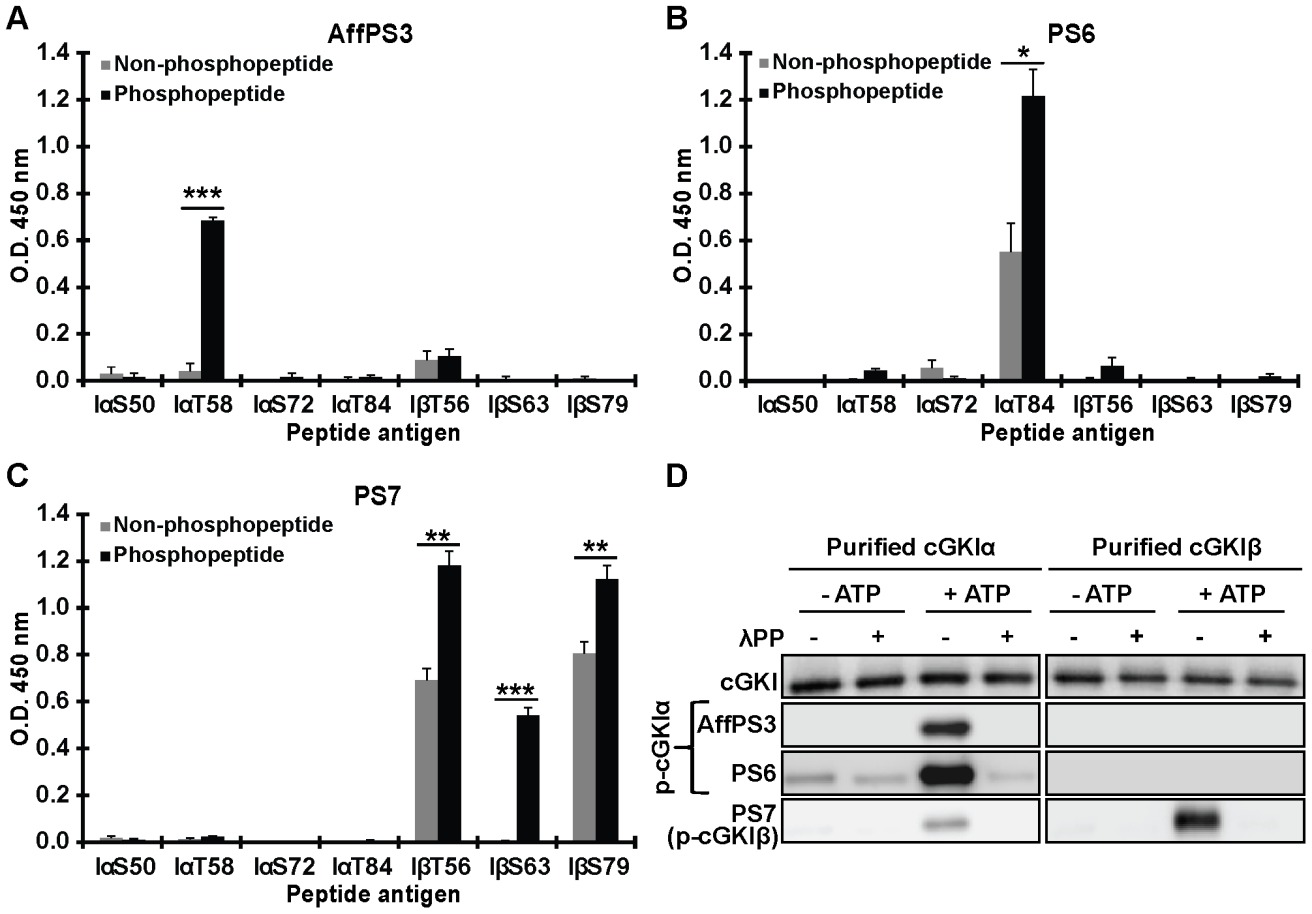
Validation of phospho-specific antisera by ELISAs with antigenic peptides (A–C) and Western blotting with purified proteins (D). Three polyclonal rabbit antisera were analyzed for their specificity and sensitivity to detect distinct phospho-sites of cGKIα (affinity-purified antiserum AffPS3, and non-purified antiserum PS6) or cGKIβ (non-purified antiserum PS7). (A–C) ELISAs were used to test binding of the antisera to non-phosphorylated (*grey bars*) and phosphorylated (*black bars*) peptide antigens (for peptide IDs and sequences, see Table 1). Data shown are means from three independent experiments ± SEM; *p≤0.05, **p≤0.01, ***p≤0.001. (D) Western blot detection of autophosphorylated cGKIα and cGKIβ by the antisera. Purified cGKI isoforms were incubated in the absence or presence of 0.1 mM ATP for 15 min at 30°C. Aliquots of the reactions were subsequently treated with lambda protein phosphatase (λPP) for 90 min at 30°C. Proteins (20 ng) were separated on SDS gels and Western blots were probed with a pan-(nonphospho-specific) cGKI antibody that detects both cGKIα and cGKIβ in their non-phosphorylated state (*upper panels*), and with the newly generated phospho-specific antisera. Data shown in D are representative for at least three independent experiments.

As depicted in Table 1, for each selected autophosphorylation site, a phospho-peptide was synthesized that contained the respective phospho-Ser or phospho-Thr residue, and 2 to 3 different peptides were pooled to immunize rabbits. The specificity of the antisera was evaluated by ELISAs with the non-phosphorylated and phosphorylated antigenic peptides as well as by Western blot analysis using purified non-phosphorylated and autophosphorylated cGKI isozymes. Polyclonal serum 3 (PS3), which recognized both phospho-Thr58 and nonphospho-Thr58 of cGKIα (data not shown), was subjected to affinity purification against the antigenic phospho-peptide, yielding affinity purified PS3 (AffPS3). Indeed, ELISA results showed that AffPS3 specifically detects the antigenic peptide containing phospho-Thr58, but not the corresponding non-phosphorylated peptide or any other of the tested peptides (Fig. 2A). In addition, two additional non-purified antisera with good specificities for phosphorylated over non-phosphorylated sites were identified by ELISAs: polyclonal serum 6 (PS6) detects phospho-Thr84 of cGKIα (Fig. 2B), and polyclonal serum 7 (PS7) detects phospho-Thr56, phospho-Ser63, and phospho-Ser79 of cGKIβ (Fig. 2C). We did not obtain antisera that recognized specifically phospho-Ser50 or phospho-Ser72 of cGKIα (data not shown).

**Figure 3:**
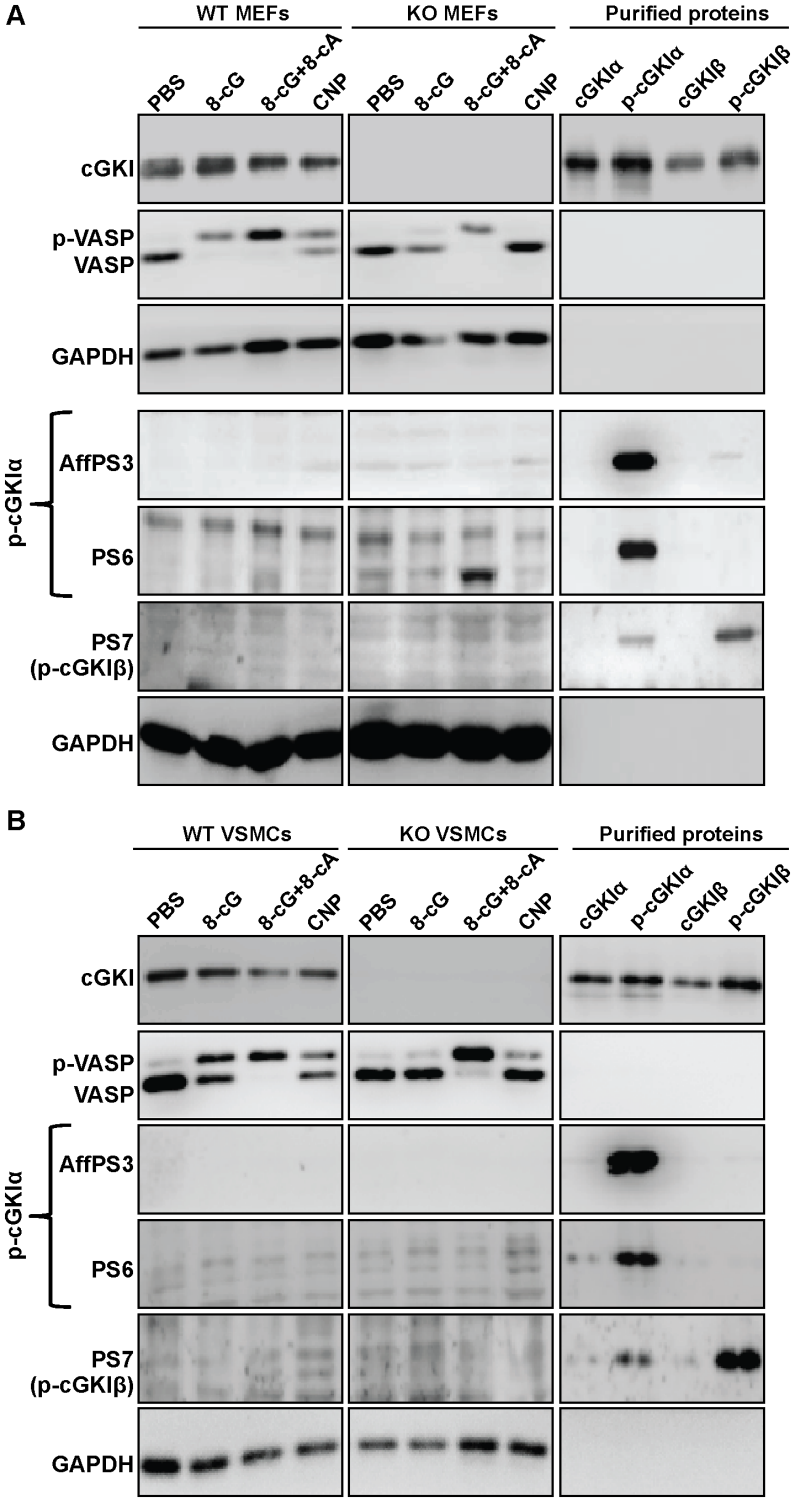
Analysis of N-terminal cGKI phosphorylation in intact MEFs (A) and VSMCs (B). Cells from wild-type (WT) and cGKI-knockout (KO) mice were incubated for 15 min at 37°C under control conditions (PBS), or in the presence of 1 mM 8-Br-cGMP (8-cG), 1 mM 8-Br-cGMP and 10 mM 8-Br-cAMP (8-cG+8-cA), or 100 nM CNP. Then, cells were lysed in denaturating buffer and cell lysates were subjected to Western blot analysis with a pan-(nonphospho-specific) cGKI antibody, anti-VASP, anti-GAPDH, and the phospho-specific antisera AffPS3, PS6, and PS7. Phosphorylation of VASP at Ser157 (p-VASP) was monitored by immunodetection of the band shift to a higher apparent molecular weight. The protein amounts of MEF lysates loaded were 15 µg for immunostaining with anti-cGKI and anti-VASP, and 50 µg for immunodetection with the phospho-specific antisera; 12 µg of VSMC lysates were loaded; GAPDH was used as respective loading control. Purified proteins (4 ng) were loaded as controls for non-phosphorylated (cGKIα, cGKIβ) and autophosphorylated (p-cGKIα, p-cGKIβ) cGKI isoforms. Similar results were obtained in three independent experiments.

Western blots with purified cGKIα and cGKIβ confirmed the isoform- and phospho-specific detection by the respective antisera (Fig. 2D). Autophosphorylation of the purified cGKI isoforms was induced by incubation with ATP (0.1 mM). The non-specific lambda protein phosphatase was added to dephosphorylate the cGKI proteins, confirming that the antibodies indeed recognized the phosphorylated epitopes. As compared to a pan-cGKI antibody [26] that detects ng-amounts of the non-phosphorylated protein (*e.g.*, 20 ng were loaded in Fig. 2D, and 4 ng were loaded in Fig. 3, *right panel* *s*), the newly generated antisera appeared to recognize phospho-cGKI species at least with the same or even higher sensitivity in the lower ng-range. In good correlation with the ELISA data (Fig. 2A–C), AffPS3 selectively detected phosphorylated cGKIα, while PS6 showed weak cross-reactivity with non-phosphorylated cGKIα. PS7 recognized predominantly the phosphorylated cGKIβ isoform, but not the non-phosphorylated protein, and showed weak cross-reactivity to phosphorylated cGKIα (Fig. 2D).

**Figure 4:**
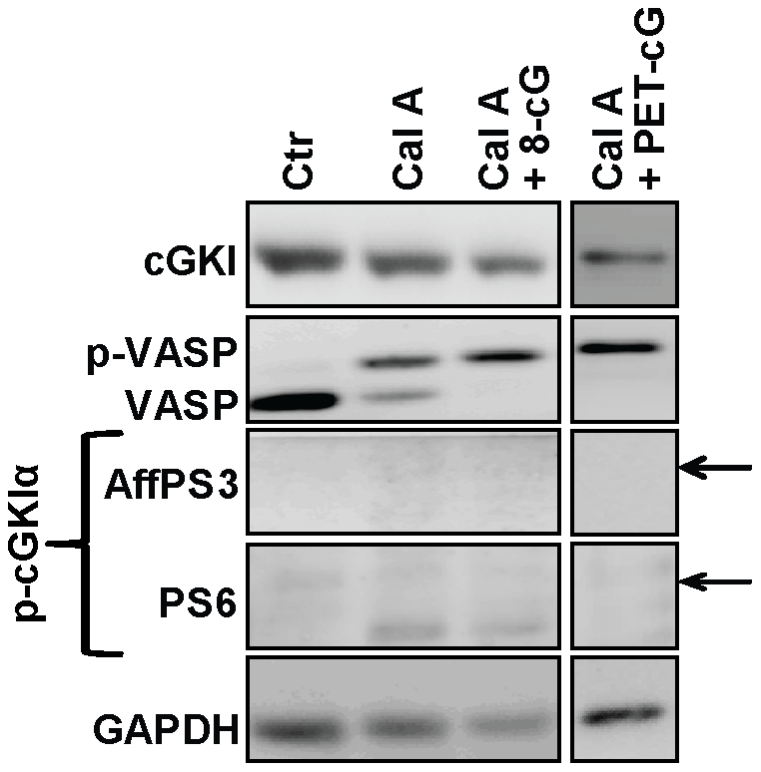
Effect of inhibition of protein Ser/Thr phosphatases on N-terminal cGKI phosphorylation in intact cells. Wild-type MEFs were incubated at 37°C under control conditions (1% DMSO in PBS for 15 min; Ctr), or for 15 min in the presence of 100 nM of the PP1/PP2A inhibitor, calyculin A (Cal A), or for 15 min in the presence of 100 nM calyculin A followed by 15 min with 1 mM 8-Br-cGMP (Cal A+8-cG) or 1 mM 8-Br-PET-cGMP (Cal A+PET-cG). Then the cells were lysed in denaturating buffer and cell lysates (10 µg) were analyzed by Western blotting with the indicated antibodies. GAPDH was used as loading control. The arrows indicate the positions expected for phospho-cGKI species as determined by co-loading of purified proteins on the same gel. Similar results were obtained in three independent experiments.

The ELISA and Western blot results indicated that we obtained three phospho-specific antisera detecting distinct autophosphorylated cGKI species with high specificity and sensitivity: AffPS3 detects phospho-Thr58 of cGKIα, PS6 detects phospho-Thr84 of cGKIα, and PS7 detects phospho-Thr56, phospho-Ser63, and phospho-Ser79 of cGKIβ (Table 2). These antisera were used for all further experiments.

**Table 2:** Phospho-sites detected by the antisera generated in this study.

Antiserum	Phospho-site detected
AffPS3	Thr58 of cGKIα
PS6	Thr84 of cGKIα
PS7	Thr56, Ser63 and Ser79 of cGKIβ

### The antisera do not detect phosphorylation of cGKI in intact mouse cells and tissues

To study cGKI autophosphorylation *in vivo*, we analyzed murine cell types and tissues that express a functional endogenous cGMP-cGKI signaling system with our phospho-specific antibodies. In the first set of experiments, cultured mouse embryonic fibroblasts (MEFs) and primary aortic vascular smooth muscle cells (VSMCs) were investigated. In general, these cells express both cGKIα and cGKIβ, but MEFs express predominantly cGKIα and VSMCs express more cGKIβ than cGKIα [24]. The status of N-terminal cGKI phosphorylation was monitored under basal conditions and after stimulation of cGKI catalytic activity in intact cells. It was expected that the latter maneuver would increase autophosphorylation of cGKI [6]. The intracellular endogenous cGKI was activated by incubation of the cells with cell-permeable 8-Br-cGMP, with 8-Br-cGMP in combination with 8-Br-cAMP, or with C-type natriuretic peptide (CNP). CNP stimulates GC-B, a particulate guanylyl cyclase, and thereby increases the intracellular cGMP concentration in both MEFs [24] and VSMCs [28]. As readout for cGKI activation, we monitored the phosphorylation of vasodilator-stimulated phosphoprotein (VASP) a known cGKI substrate. VASP can be phosphorylated on Ser157 by multiple kinases including cGKIα, cGKIβ, and cAMP-dependent protein kinase [29], [30]. Phospho-VASP (Ser157) migrates at an apparent higher molecular weight compared to dephospho-VASP, which is conveniently detected on Western blots with VASP antibodies. Note that cells and tissues were lysed in buffer containing SDS and phosphatase inhibitors to block potential dephosphorylation of proteins during sample preparation.

As shown in Fig. 3A (*upper panels*), VASP phosphorylation was induced in wild-type MEFs by treatment with 8-Br-cGMP, 8-Br-cGMP and 8-Br-cAMP, or CNP for 15 min at 37°C. In cGKI-deficient MEFs, 8-Br-cGMP and CNP did not induce VASP phosphorylation, confirming that the cGMP-induced VASP phosphorylation observed in wild-type cells was indeed mediated by cGKI. Combined treatment with 8-Br-cGMP and 8-Br-cAMP still resulted in an increase of phospho-VASP in cGKI-deficient MEFs, most likely because 8-Br-cAMP had activated the cAMP-dependent protein kinase. Surprisingly, although our stimulation protocol clearly induced cGKI phosphotransferase activity in intact MEFs, our phospho-specific cGKI antisera did not detect epitopes on the Western blot that could potentially represent autophosphorylated cGKI species, both under basal and stimulated conditions, and when huge amounts of protein (50 µg) were loaded (Fig. 3A, *lower panels*). Very weak signals were detected by AffPS3 and PS7, and stronger bands were detected by PS6. However, it was unlikely that these signals were derived from phosphorylated cGKI. First, most of these bands did not co-migrate with purified autophosphorylated cGKI. Second, similar signals were detected under basal and cGKI-activated conditions. Third, and most importantly, these bands were also detected in cGKI-deficient MEFs. A similar experiment was performed with primary VSMCs (Fig. 3B). Again, although activation of cGKI was confirmed by an increase in VASP phosphorylation, the phospho-cGKI antisera did not detect any specific signals in intact cells.

To rule out the possibility that the failure to detect phospho-cGKI in these experiments was due to a transient existence of the phosphorylated species, two additional experiments were performed with wild-type MEFs. First, in a time-course experiment cells were incubated with 8-Br-cGMP or with a combination of 8-Br-cGMP and 8-Br-cAMP at 37°C and aliquots were analyzed for phospho-cGKI after 1, 2, 4, 8, 16, and 32 min. As monitored by VASP phosphorylation, cGKI was already active 1 min after addition of 8-Br-cGMP. However, the phospho-specific antisera did not detect any phospho-cGKI species in the interval between 1 and 32 min (data not shown). Next, we tested whether phospho-cGKI could be detected when the experiment was performed in the presence of the cell-permeable protein phosphatase inhibitor calyculin A, which inhibits the Ser/Thr phosphatases PP1 and PP2A [31]. As illustrated in Fig. 4, incubation of intact MEFs with calyculin A alone resulted in an increased level of phospho-VASP indicating that the compound did indeed block protein dephosphorylation in intact cells. Additional incubation with 8-Br-cGMP or with the more lipophilic cGMP analogue, 8-Br-PET-cGMP, further increased VASP phosphorylation confirming that cGKI was activated. However, even under phosphatase inhibition, we could not detect any phospho-cGKI species (Fig. 4). Similar results were obtained when cells were incubated with calyculin A and a combination of 8-Br-cGMP and 8-Br-cAMP (data not shown).

**Figure 5:**
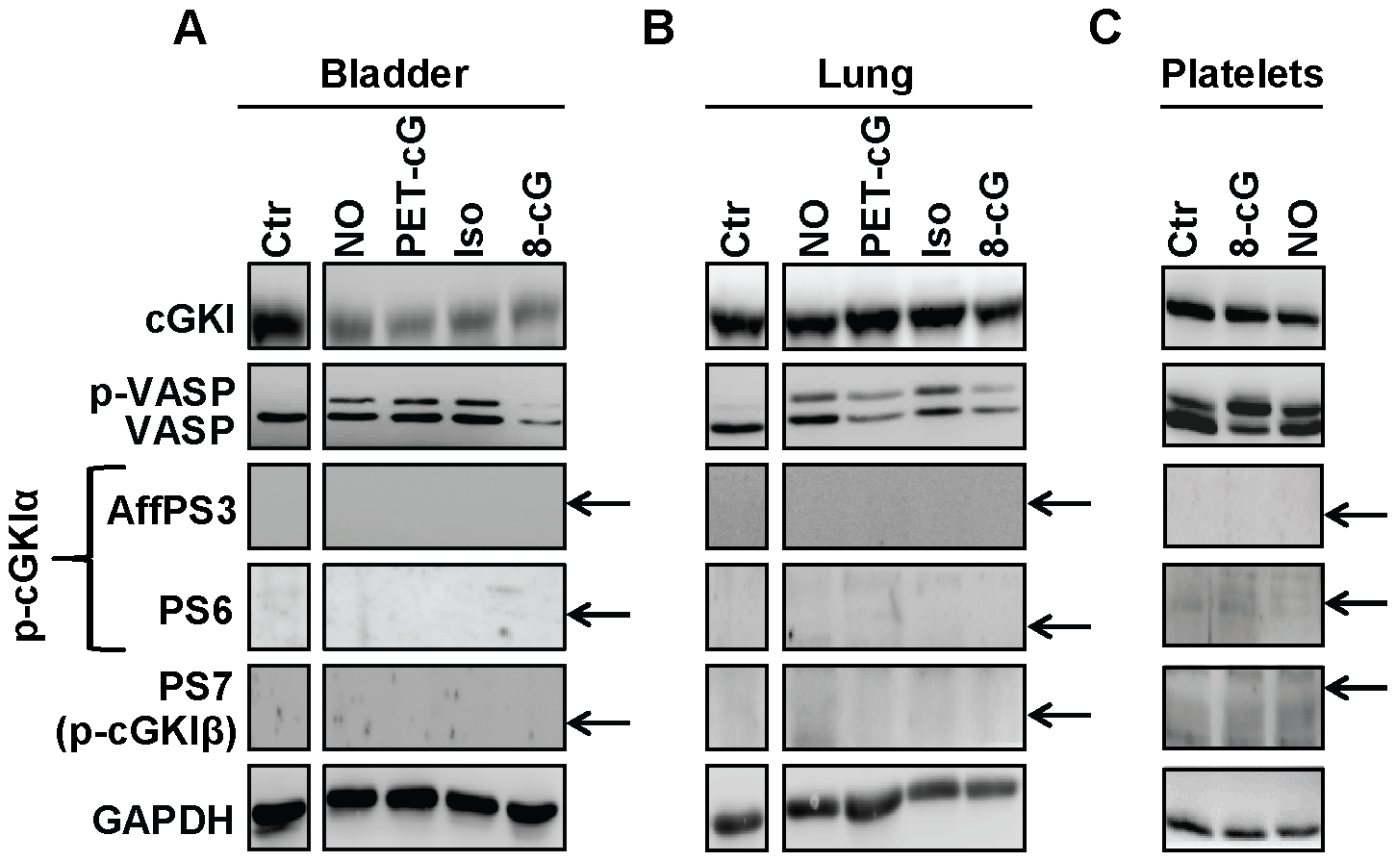
Analysis of N-terminal cGKI phosphorylation in native mouse tissues and platelets. (A) Bladder and (B) lung were rapidly isolated from wild-type mice and then incubated in Tyrode buffer for 15 min at room temperature under control conditions (Ctr) or in the presence of 100 nM calyculin A and 0.1 mM DEA-NONOate (NO), 1 mM 8-Br-PET-cGMP (PET-cG), 0.01 mM isoprenaline hydrochloride (Iso), or 1 mM 8-Br-cGMP (8-cG). (C) Platelets were isolated from wild-type mice and incubated for 10 min at 37°C under control conditions (Ctr) or in the presence of 1 mM 8-Br-cGMP (8-cG) or 3 mM DEA-NONOate (NO). Lysates (22 µg for bladder, 30 µg for lung, and equal fractions by volume for platelets) were subjected to Western blot analysis with the indicated antibodies. GAPDH was used as loading control. The arrows indicate the positions expected for phospho-cGKI species as determined by co-loading of purified proteins on the same gel. The displayed results are representative for three independent experiments.

The analyses of N-terminal phosphorylation of cGKI isoforms were so far performed with cultured cells, leaving the possibility that phospho-cGKI exists in native cells and tissues. Therefore, we analyzed acutely isolated mouse tissues with our phospho-specific cGKI antibodies. The following tissues were analyzed: aorta, which expresses cGKIα and cGKIβ; bladder and platelets, which express predominantly cGKIβ; and lung and cerebellum, which express predominantly cGKIα [4]. The specificity of weak potential phospho-cGKI signals detected in wild-type tissues was evaluated by comparison with purified autophosphorylated cGKI as well as with non-specific signals detected in tissue extracts of cGKI-deficient mice. None of our antisera detected specific phospho-cGKI signals in the freshly isolated tissues (data not shown). It was possible that the level of phosphorylated cGKI *in vivo* is very low under basal conditions, but increases to detectable levels after stimulation of cGKI activity. To test this hypothesis, we treated acutely isolated bladder and lung tissues in the presence of calyculin A with 8-Br-cGMP, 8-Br-PET-cGMP, or DEA-NONOate, a NO-releasing compound that promotes endogenous cGMP synthesis via soluble guanylyl cyclase. Although these treatments increased the level of phospho-VASP indicating that cGKI was activated, phosphorylated cGKI species were not detectable with our antisera in the stimulated tissues (Fig. 5A, B). To evaluate the possibility of cAMP-induced phosphorylation of cGKI, freshly islotated tissues were incubated with calyculin A and isoprenaline, which increases the intracellular cAMP concentration. Again, no phospho-cGKI species could be detected (Fig. 5A, B). Negative results were also obtained with mouse platelets that were stimulated with 8-Br-cGMP or DEA-NONOate (Fig. 5C). Finally, antisera AffPS3 and PS6 were also tested on tissue sections of the cerebellum that contains cGKIα-positive Purkinje cells [32], but no specific phospho-cGKI signals were detected (data not shown).

Taken together, our persistent attempts to detect N-terminally phosphorylated cGKI species in intact cells and tissues *in vivo* under basal or cGKI-activated conditions were unsuccessful.

### Under *in vitro* conditions, autophosphorylation of cGKI is preferred as compared to phosphorylation of exogenous substrates

**Figure 6:**
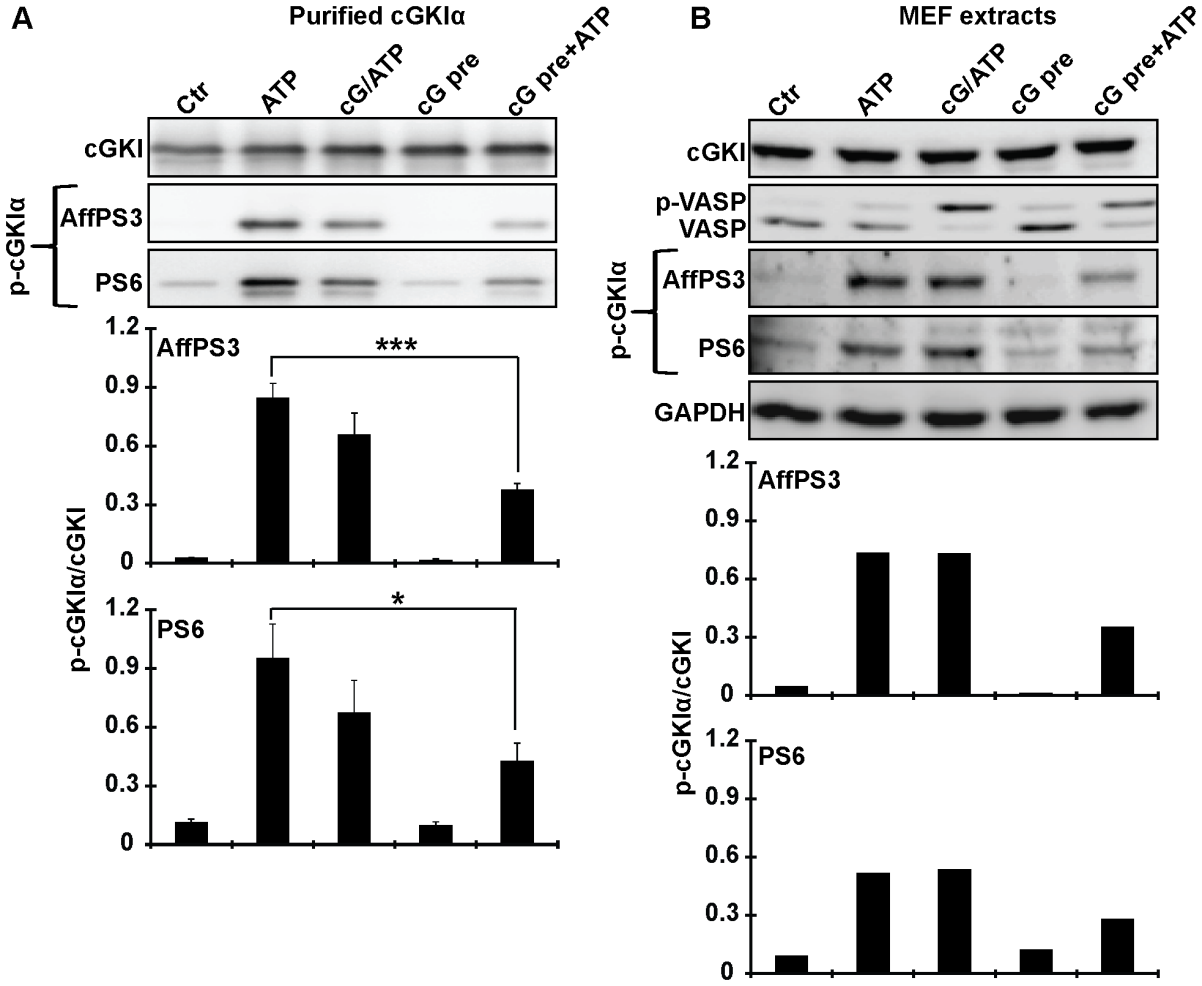
N-terminal phosphorylation of cGKI in purified preparations (A) and cell extracts (B). (A) Purified cGKIα or (B) cell extracts prepared from wild-type MEFs in non-denaturating buffer were incubated for 15 min at 30°C under control conditions (Ctr) or in the presence of 0.1 mM ATP or 0.1 mM ATP combined with 0.1 mM cGMP (cG/ATP). Alternatively, samples were pre-incubated for 15 min at 30°C with 0.1 mM cGMP. Then they were further incubated either under control conditions without ATP (cG pre) or in the presence of 0.1 mM ATP (cG pre+ATP) for another 15 min at 30°C. Purified proteins (20 ng) or cell extracts (10 µg) were analyzed for N-terminal phosphorylation of cGKIα by Western blotting with antisera AffPS3 and PS6. The total amount of cGKI was detected with a pan-(nonphospho-specific) cGKI antibody, and phospho-VASP in cell extracts was monitored with anti-VASP antibody. GAPDH was used as loading control for cell extracts. Below the Western blots, the semiquantitative densitometric analysis of phospho-cGKI signals is shown. It was performed using ImageJ software [37] and is given as the ratio of the intensity of the phospho-band detected by AffPS3 or PS6 (p-cGKI) divided by the intensity of the respective cGKI band detected by the pan-cGKI antibody in the same sample (cGKI). Data shown in the bar graphs in (A) are means ± SEM (n = 3 independent experiments); *p≤0.05, ***p≤0.001. Data shown in the bar graphs in (B) are means of 2 independent experiments.

To get more insights into the apparent difference in cGKI autophosphorylation *in vitro* versus *in vivo*, and to evaluate the utility of the new phospho-cGKI antibodies as experimental tools, we performed further experiments with purified proteins and broken-cell preparations. Purified cGKIα was incubated with ATP alone, cGMP and ATP simultaneously, cGMP alone, or it was first pre-incubated with cGMP before ATP was added. Autophosphorylated cGKIα was then detected with antisera AffPS3 and PS6 (Fig. 6A). As expected (Fig. 2D), addition of ATP alone was sufficient to induce efficient autophosphorylation. Interestingly, compared to ATP alone both phospho-specific antisera detected a decreased level of phospho-cGKIα in the presence of cGMP, particularly after pre-incubation of the enzyme with cGMP (Fig. 6A). In contrast, pre-incubation of purified cGKIβ with cGMP did not significantly affect its autophosphorylation (data not shown) suggesting that inhibition of autophosphorylation by cGMP is a specific property of the cGKIα isoform.

ATP-induced phosphorylation of endogenous cGKIα and its inhibition by cGMP were also observed when cell extracts prepared from MEFs were incubated *in vitro* with the respective compounds (Fig. 6B). This experiment showed also that our antibodies can be used for the specific detection of phospho-cGKIα species in complex protein mixtures such as cell extracts. Importantly, a comparison of the levels of phospho-VASP and phospho-cGKIα in the presence of ATP alone (Fig. 6B, *lane 2*) indicated that, at least in *in vitro*, (a) cGKIα can undergo N-terminal phosphorylation in a non-cGMP-activated state and (b) autophosphorylation alone does not activate heterophosphorylation of substrate proteins like VASP.

## Discussion and Conclusions

In this study, phospho-specific antibodies were generated that detect autophosphorylated cGKIα and cGKIβ with high specificity and sensitivity. Autophosphorylation of cGKI could be induced in purified preparations or cell extracts by addition of ATP. However, phospho-cGKI species could not be detected in intact cells and tissues, both under basal conditions and after induction of cGKI catalytic activity. These findings challenge the physiological relevance of the current cGKI activation model that is based on *in vitro* experiments with purified cGKI. According to this model (Fig. 1B) [6], autophosphorylation is a preferential process as compared to phosphorylation of exogenous substrates. It can be stimulated by cGMP or cAMP and increases the basal catalytic activity of cGKI even after release of the activator. Thus, one would expect that intact tissues contain cGKI in both phosphorylated and non-phosphorylated forms. In contrast to this model, the results of the present study with intact cells and tissues indicate that neither cGKIα nor cGKIβ is phosphorylated in its N-terminal region *in vivo*, at least at the residues detected by our antisera. Even under conditions that stimulate the catalytic activity of cGKI, no autophosphorylation could be detected. Our phospho-specific antisera detected two of four major *in vitro*-autophosphorylation sites of cGKIα (phospho-Thr58 and phospho-Thr84) and three potential phospho-sites of cGKIβ (phospho-Thr56, phospho-Ser63, and phospho-Ser79). We cannot exclude phosphorylation of cGKI at Ser/Thr residues that were not recognized by our antibodies. Alternative *in vivo* phosphorylation sites of cGKI under baseline and activating conditions could be identified by a hypothesis-free approach based, for instance, on mass spectrometry. However, autophosphorylation of these alternative sites would be expected to coincide with autophosphorylation of at least some of the major sites that were detected by our antisera.

Our failure to demonstrate N-terminal autophosphorylation of cGKI in intact cells with phospho-specific antibodies is in agreement with previous studies that used other methods to determine the *in vivo*-phosphorylation status of cGKI. Hou et al. [33] studied the phosphorylation of cGKIα in transfected HEK-293 cells loaded with [^32^P]PP_i_ and could not detect an increase of [^32^P]-cGKIα after treatment of the cells with 8-Br-cGMP. Pinske et al. [12] determined the phosphorylation state of purified bovine lung cGKIα by mass spectrometry. The enzyme was completely phosphorylated on Thr516, but no major phosphorylation site was detected in the N-terminal region under basal conditions. Thr516 is localized in the activation loop within the catalytic core of the protein kinase, and its phosphorylation is essential for enzymatic activity [20]. In line with the mass spectrometric data, purified cGKI contains 1.1–1.4 mol phosphate/mol subunit [11]. Together, the results of the present and previous studies strongly suggest that cGKI occurs *in vivo* as a phosphoprotein that is mainly phosphorylated at Thr516, but shows no N-terminal autophosphorylation.

Our experiments with purified proteins (Figs. 2D and 6A) and broken-cell preparations (Fig. 6B) confirmed that cGKI autophosphorylates its N-terminal region *in vitro*. Phosphorylation of cGKI could be induced by addition of ATP (0.1 mM) to the purified enzymes or cell extracts. Interestingly, ATP-induced autophosphorylation of cGKIα was inhibited by pre-incubation with saturating amounts of cGMP (0.1 mM). These data are in line with previous studies that reported cGKI autophosphorylation in the presence of ATP alone and its inhibition by the addition of cGMP [8], [9]. Indeed, a recent analysis of the interactions of cGKIα with cGMP and ATP by mass spectrometry showed that ATP can enter the ATP-binding site already in the basal state of cGKI in the absence of cGMP, and that the cGKI-ATP interaction is weakened in the cGMP-activated conformation of the kinase [34]. The apparent discrepancy of these results with other studies reporting that cGKI autophosphorylation can be stimulated by cGMP [5], [6] might be explained by different cGMP concentrations that were used in the respective autophosphorylation reactions. High and low cGMP concentrations might induce different protein conformations that hinder or boost autophosphorylation, respectively [35], [36]. Another interesting finding of our study was that addition of ATP alone led to efficient cGKI phosphorylation in cell extracts without an apparent increase in phosphorylation of the cGKI substrate, VASP (Fig. 6B, *lane 2*). Taken together, our data indicate that N-terminal phosphorylation of cGKI (a) does not require, and can be even inhibited by a cGMP-activated conformation of the kinase and (b) does not increase the basal catalytic activity of the kinase toward exogenous substrates in the absence of cGMP.

Why does cGKI readily autophosphorylate *in vitro* but not *in vivo*? Considering that purified cGKI autophosporylates in the presence of 0.1 mM ATP, and that the intracellular ATP concentration is typically 1–10 mM, one would expect that autophosphorylated cGKI occurs *in vivo* already under basal conditions. However, we did not detect phospho-cGKI in intact cells. This suggests that the conformation and/or environment of the kinase in intact cells differ fundamentally from purified protein and broken-cell preparations, in which autophosphorylation occurred. The balance between auto- and heterophosphorylation could be influenced by the availability of physiological partner proteins of cGKI, such as anchoring and substrate proteins. Purified cGKI preparations lack these factors and cell extracts contain them in much lower concentrations than intact cells. Interestingly, cell extracts showed cGKI autophosphorylation in the absence of VASP phosphorylation (Fig. 6B, *lane 2*), whereas intact cells demonstrated VASP phosphorylation in the absence of autophosphorylation (Figs. 3, 4, 5). Thus, it appears that under *in vitro* conditions autophosphorylation is preferred as compared to phosphorylation of exogenous substrates. However, autophosphorylation is obviously prevented in intact cells by the interaction of cGKI with other proteins, and after cGMP activation only heterophosphorylation of substrate proteins occurs. This also implies that autophosphorylation is not involved in cGKI activation *in vivo*, and we propose to revise the working model of cGKI accordingly (Fig. 1B). The finding that cGKI is most likely not N-terminally autophosphorylated in intact cells does also inform screening strategies aiming to identify novel cGKI-binding drugs based on *in vitro* assays with purified cGKI protein. Contrary to what would be suggested by the previous model that incorporated autophosphorylated cGKI as a relevant enzyme species, our present results strongly suggest that these assays should not be performed with autophosphorylated cGKI.

In conclusion, this study provides important new insights into the structure-function relationship of cGKI in intact cells. Although readily induced *in vitro*, autophosphorylation of cGKIα and cGKIβ does most likely not occur *in vivo*. Thus, the catalytic activity of cGKI in intact cells appears to be independent of N-terminal autophosphorylation. These findings also support the general notion that the *in vitro*- and *in vivo*-biochemistry of a given protein can be fundamentally different.
